# Development, implementation, and prospective validation of a model to predict 60-day end-of-life in hospitalized adults upon admission at three sites

**DOI:** 10.1186/s12911-020-01235-6

**Published:** 2020-09-07

**Authors:** Vincent J. Major, Yindalon Aphinyanaphongs

**Affiliations:** Department of Population Health, NYU Langone Health, 227 East 30th St, 6th Floor, New York, NY 10016 USA

**Keywords:** Mortality prediction, Palliative care, Supportive care, End-of-life care, Advance directives, Medical informatics, Machine learning, Electronic health records

## Abstract

**Background:**

Automated systems that use machine learning to estimate a patient’s risk of death are being developed to influence care. There remains sparse transparent reporting of model generalizability in different subpopulations especially for implemented systems.

**Methods:**

A prognostic study included adult admissions at a multi-site, academic medical center between 2015 and 2017. A predictive model for all-cause mortality (including initiation of hospice care) within 60 days of admission was developed. Model generalizability is assessed in temporal validation in the context of potential demographic bias. A subsequent prospective cohort study was conducted at the same sites between October 2018 and June 2019. Model performance during prospective validation was quantified with areas under the receiver operating characteristic and precision recall curves stratified by site. Prospective results include timeliness, positive predictive value, and the number of actionable predictions.

**Results:**

Three years of development data included 128,941 inpatient admissions (94,733 unique patients) across sites where patients are mostly white (61%) and female (60%) and 4.2% led to death within 60 days. A random forest model incorporating 9614 predictors produced areas under the receiver operating characteristic and precision recall curves of 87.2 (95% CI, 86.1–88.2) and 28.0 (95% CI, 25.0–31.0) in temporal validation. Performance marginally diverges within sites as the patient mix shifts from development to validation (patients of one site increases from 10 to 38%). Applied prospectively for nine months, 41,728 predictions were generated in real-time (median [IQR], 1.3 [0.9, 32] minutes). An operating criterion of 75% positive predictive value identified 104 predictions at very high risk (0.25%) where 65% (50 from 77 well-timed predictions) led to death within 60 days.

**Conclusion:**

Temporal validation demonstrates good model discrimination for 60-day mortality. Slight performance variations are observed across demographic subpopulations. The model was implemented prospectively and successfully produced meaningful estimates of risk within minutes of admission.

## Background

### Supportive and palliative care

Supportive care describes a myriad of interventions intended to prevent or improve symptoms of disease or side-effects of treatment. Patients with terminal illness often receive supportive care as their disease progresses towards palliative and end-of-life care. Unfortunately, many patients do not receive palliative care until their last weeks of life [1] despite guidelines recommending palliative care for any patient diagnosed with a chronic or serious illness that will ultimately lead to their death [2]. Practical methods to identify patients who would benefit from palliative and, more generally, supportive care and end-of-life planning are needed [3].

Physicians make treatment decisions—including whether to initiate or defer palliative care—based upon their perception of a patient’s condition. Unfortunately, physicians tend to be optimistic when estimating prognosis [4–6]. Since the entire process relies on human judgement, patients who have previously been overlooked for supportive care can continue to slip through the cracks. Automated systems can augment clinician gestalt as a failsafe mechanism to improve quality and consistency of care.

### Mortality prediction

Many systems have been developed to estimate mortality risk. Early methods developed scores to be applied, by hand, at the bedside with a small number of parameters. Promising recent works apply machine learning to predict mortality risk upon admission, or shortly afterwards, to prompt palliative care [7–9]. These works rely on high-level administrative data [9], claims data [10], billing codes from the electronic health record (EHR) [7, 11], or concepts extracted from clinical notes [8]. Many of these works focus on long-term mortality, typically 1-year, or restrict to specific cohorts or datasets that limit their utility to influence care decisions. Although many models have been developed, few machine learning systems are implemented in clinical practice [12]. Even fewer studies have assessed model safety and performance across sites.

## Objective

To develop and validate a machine learning model to predict short-term mortality at the instant of inpatient admission using EHR data. Model validation consists of two steps: first, model generalization is investigated by assessing testing set performance across sites. Second, the model is implemented and prospectively validated to assess technical feasibility and real-world performance before release into the EHR.

## Methods

### Data

#### Study setting

This prognostic study was conducted at NYU Langone Health, a multi-site academic medical center in New York City. At the time of model development, July 2018, NYU Langone Health consisted of approximately 1300 beds across one general and one Orthopedics hospital in the borough of Manhattan and one general hospital in Brooklyn.

This project met the definition of quality improvement outlined by the NYU Grossman School of Medicine IRB and is not considered human subjects research and did not require IRB approval. This study follows the reporting guidelines set out in the Transparent Reporting of a Multivariable Prediction Model for Individual Prognosis or Diagnosis (TRIPOD) statement.

#### Patient population

A retrospective dataset was selected by identifying all adults hospitalized between January 1, 2015 and December 31, 2017 (> 18 years old at admission). Admissions for inpatient hospice care were excluded along with observation stays but patients ‘boarding’ in the emergency department were included. No other inclusion criteria were imposed.

#### Mortality outcomes

All-cause death outcomes in the community can be problematic for predictive modeling as patients can die anywhere and reporting of deaths can vary widely. One of the primary challenges is missingness that can be caused by a variety of practical, social and technical reasons. Researchers often improve their data by combining several sources of data into a composite [13]. We follow this trend and exploit three available sources: 1) internal system-wide death data, 2) purchased death data (derived from the Social Security Administration’s Master Death File), and 3) hospice discharge disposition data (both inpatient and home hospice). None of these mechanisms perfectly capture all deaths but, together, establish a measure of ‘end-of-life’ where the addition of hospice improves robustness but adds noise (eResults and eFigure 1).

After extensive discussion with physicians, a primary outcome of mortality within 60 days of admission was selected. The rationale for 60 days is to promote urgency in end-of-life decision-making while allowing sufficient time to initiate supportive and palliative care interventions, both during the hospitalization and in the community post-discharge. At the time of model development (July 2018), more than 6 months had passed since the end of the study period ensuring adequate time for 60-day outcomes to accrue.

#### Feature construction

To estimate risk within minutes of admission, all predictors must be reliably accessible at that time (i.e. data collected after arrival likely cannot be used). Instead, one year of historical data (up to the day prior) is considered for each admission. Patient demographics and discrete data describing prior encounters are collected along with several categories of coded data used in related works [7, 10, 11], namely: ICD-10 (International Classification of Diseases) diagnosis codes, CPT (Current Procedural Terminology) procedure codes, RxNorm medication codes, and LOINC (Logical Observation Identifiers Names and Codes) laboratory result codes. Each data type, except demographics, are dated in a patient’s history and can occur many times.

Features are constructed from these data similarly to related works [7, 10, 11]. Specifically, each patient’s history is segmented into four time slices with boundaries at 30, 90, and 180 days preceding admission [7], excluding all data collected more than a year prior. Each data category (e.g. ICD-10 diagnoses) is aggregated in each slice into:

- Count of each unique code,
- Count of unique codes and total code count across days, and
- Mean, variance, minimum, maximum, and range of the daily number of codes.

While a typical patient may have fewer than a dozen unique ICD-10 codes, all patients have aggregate values, e.g. total codes during slice and max number of daily codes. With these features, a model may learn differences in specific disease types as well as disease burden and utilization with these features.

### Experimental design

**Fig. 1:**
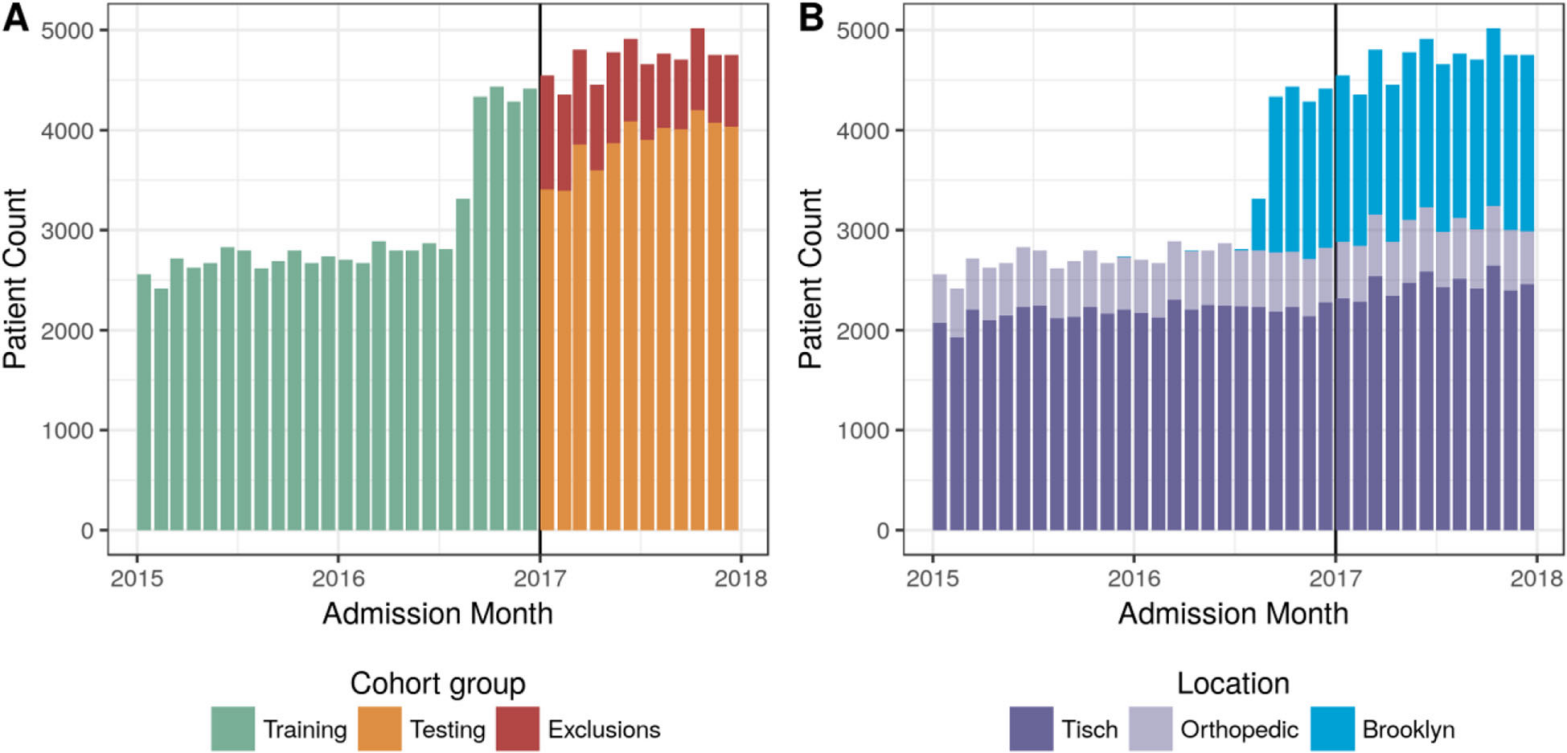
Monthly admissions stratified by **a**) model development cohort and **b**) hospital location

A retrospective cohort-study experimental design that ‘enrolls’ each admission is employed with a temporally separated testing set. This design has been demonstrated in prior work [14] to improve implementation performance without overestimation during validation. Three years of data were partitioned into training and testing cohorts on January 1, 2017 resulting in 24 and 12 months respectively as described in Fig. 1a. No other cohort selection criteria were applied during the training period—all (re)admissions were included. Patients who were readmitted within the testing period (2017) following their ‘enrollment’ during the training period (2015/2016) are excluded to ensure no individual patient is present in both groups.

A dramatic increase in monthly admissions is evident in Fig. 1 as the NYU Langone Brooklyn hospital transitioned onto the existing EHR system in August 2016. The training cohort therefore underrepresents Brooklyn patients with only 4.5 months of data included (19% from 24 months). This non-random underrepresentation during model derivation is a blatant example of a wider challenge faced by any predictive model: generalizing into a future population.

### Model development

Many thousands of candidate predictors are expected where only a small fraction exist for a typical patient. With this many predictors, overfitting to spurious associations and small samples is difficult to avoid. Predictors are pruned by requiring at least once occurrence in both outcome groups (i.e. survival and death) and a total count exceeding 100. This leaves 9614 features (11% of 87,226) for modeling. Some training cohort patients (19.6%) are left with only demographic features and are removed from model training. No data imputation is performed.

Many of these features are sparse (e.g. specific ICD-10 codes), others are complete but highly nonlinear (e.g. count of procedure codes) which complicate modeling. An algorithm is needed that can learn which features—and which values within those features—are prognostic. Three classifiers were considered (eMethods): logistic regression with lasso regularization [15], XGBoost [16], and random forest [17]. The later two are tree-based algorithms, known for their consistent performance on a variety of datasets [18] and within similar mortality work [10, 19]. Parameter selection was conducted within 5-fold cross validation by sampling patients (not admissions [20]) before retraining one model on the entire training set (eMethods). Model performance within the testing set is assessed with area under the receiver operating characteristic (AUROC) and area under the precision recall curve (AUPRC). To evaluate the impact of Brooklyn underrepresentation during training, testing set performance is compared across locations by considering principles of model transparency [21] and model fairness [22] (eMethods).

### Implementation

#### Operating threshold

Before implementation, an operating threshold for intervention must be selected (eMethods). Clinical stakeholders selected a criterion of 75% positive predictive value (PPV), corresponding to one false positive from four high-risk predictions. The 75% PPV criterion was imposed on the testing set under bootstrap conditions to calculate a robust threshold (eMethods).

#### Prospective validation

In order to assess the model’s utility to influence care decisions, it was implemented and prospectively applied to patient data in a controlled manner. A ‘silent-live’ phase allowed prospective data to be collected but predictions were withheld from clinicians until initial results were assessed and any technical issues resolved. This interim period provides time to develop a clinical workflow.

After a successful silent-live period, the model was deployed *live* where it would deliver estimates of risk in near real-time to enable prospective validation. Data spanning nine months between October 2018 and June 2019 was used for analysis. During this period, all new inpatient admissions were collected but predictions were never made for children, nor those with no prior data (i.e. only demographics). For this prospective cohort study, patient data was collected to enable analysis of prediction volume, timeliness, and PPV when implemented live. Patient outcomes were only collected for patients identified at high-risk.

### Evaluation in the context of potential demographic Bias

Model fairness is an increasingly important factor affecting deployment of predictive models, especially in applications involving vulnerable populations. Model fairness is closely related to how a model generalizes across populations—particularly demographic groups—and impacts patient safety. Several recent works have reported that the explicit removal of ‘sensitive’ data elements—such as gender or race—may perpetuate inequalities observed within the data [22, 23]. In the interest of transparency [21] (one pillar of responsible machine learning), model performance in different strata of sensitive demographics is investigated (eMethods, eResults, eFigures 5 and 6). One model, trained on the entire training set is applied to sub-cohorts of the testing cohort by combinations of sensitive demographics (e.g. Black women admitted in Brooklyn) and various measures of model performance are reported. This procedure is repeated for a second model where all sensitive demographics are excluded or ‘masked’ during training (eMethods and eResults).

## Results

### Patient cohort and outcomes

In the three calendar years considered, 128,941 inpatient admissions occurred across the three hospitals including 94,733 unique patients. The population is mostly white (61%) and female (60%). Patient demographics with location, comorbidity, and outcome characteristics are reported in Table 1. The underrepresentation of Brooklyn patients in the training cohort and considerable structural differences between locations (eTable 1) lead to differences when comparing training and testing sets. Of all admissions, 4.2% led to death or hospice within 60 days and the median time from admission to outcome is 53 days with no drastic differences by cohort or demographics (eFigure 2).

**Table 1:** Demographics, outcome, comorbidity, and model predictor characteristics of the model development population

		All Patients *n* = 128,941	Training Set *n* = 72,437	Testing Set *n* = 46,458	
**Demographics** ^**a**^					
Measure	Value				
Age		% (n)	% (n)	% (n)	*
*18–29*		11.5% (14786)	10.7% (7778)	13.1% (6087)	
*30–39*		17.5% (22607)	18.0% (13053)	18.0% (8361)	
*40–49*		9.45% (12183)	9.49% (6877)	9.69% (4504)	
*50–59*		13.3% (17204)	13.5% (9784)	13.4% (6206)	
*60–69*		18.2% (23500)	18.7% (13556)	17.3% (8026)	
*70–79*		15.8% (20388)	15.8% (11439)	15.1% (7008)	
*80–89*		10.7% (13839)	10.5% (7588)	10.2% (4748)	
*90+*		3.44% (4434)	3.26% (2362)	3.27% (1518)	
Ethnicity ^b^		% (n)	% (n)	% (n)	*
*Hispanic*		9.75% (3467)	9.77% (2336)	8.62% (666)	
*Not Hispanic*		90.3% (32086)	90.2% (21584)	91.4% (7060)	
*Unknown*		-- (93388)	-- (48517)	-- (38732)	
Race		% (n)	% (n)	% (n)	*
*Black*		10.9% (14033)	11.0% (7933)	10.7% (4987)	
*East Asian*		7.38% (9520)	6.50% (4707)	9.10% (4230)	
*West Asian*		1.66% (2146)	1.68% (1219)	1.74% (807)	
*White*		61.6% (79424)	64.1% (46404)	57.3% (26642)	
*Other*		16.4% (21181)	14.8% (10692)	18.8% (8714)	
*Unknown*		2.05% (2637)	2.05% (1482)	2.32% (1078)	
Sex		% (n)	% (n)	% (n)	
*Female*		60.1% (77478)	60.3% (43664)	60.5% (28130)	
*Male*		39.9% (51459)	39.7% (28770)	39.4% (18327)	
*Unknown*		0% (4)	0% (3)	0% (1)	
Site		% (n)	% (n)	% (n)	*
*Tisch*		63.4% (81807)	72.3% (52398)	49.2% (22877)	
*Orthopedic*		15.6% (20137)	18.1% (13122)	12.8% (5938)	
*Brooklyn*		20.9% (26997)	9.55% (6917)	38% (17643)	
**Outcomes** ^**c**^		% (n)	% (n)	% (n)	
Any known death		7.93% (10229)	9.00% (6521)	5.20% (2414)	*
60-day death		4.15% (5356)	4.05% (2935)	3.57% (1657)	*
		Median [IQR]	Median [IQR]	Median [IQR]	
Days from admission to death		53 [6, 205]	83 [12, 306]	21 [1, 92.75]	*
**Comorbidities** ^**d**^		Median [IQR]	Median [IQR]	Median [IQR]	
Charlson Score		1 [0, 2]	1 [0, 2]	0 [0, 2]	*
		% (n)	% (n)	% (n)	
AIDS/HIV		0.626% (635)	0.61% (349)	0.506% (176)	
Cancer (any malignancy)		16.8% (17094)	18.2% (10432)	13.2% (4594)	*
Cerebrovascular disease		10.0% (10149)	9.99% (5716)	8.13% (2826)	*
Chronic obstructive pulmonary disease		17.9% (18218)	18.6% (10649)	13.5% (4703)	*
Congestive heart failure		12.0% (12144)	11.8% (6774)	8.56% (2978)	*
Dementia		3.67% (3721)	3.18% (1819)	3.09% (1075)	
Diabetes with chronic complications		6.34% (6439)	4.9% (2806)	5.68% (1977)	*
Diabetes without chronic complications		16.8% (17019)	16.2% (9256)	14.4% (4995)	*
Hemiplegia or paraplegia		2.92% (2962)	2.83% (1617)	2.35% (817)	*
Metastatic solid tumor		6.02% (6115)	6.39% (3657)	4.55% (1584)	*
Mild liver disease		6.40% (6495)	6.23% (3566)	5.14% (1787)	*
Moderate or severe liver disease		1.62% (1642)	1.59% (910)	1.11% (385)	*
Myocardial infarction		9.73% (9874)	9.48% (5423)	6.9% (2400)	*
Peptic ulcer disease		1.84% (1871)	1.76% (1009)	1.27% (443)	*
Peripheral vascular disease		13.1% (13278)	13.0% (7446)	9.97% (3469)	*
Renal disease		10.9% (11093)	10.4% (5937)	7.93% (2759)	*
Rheumatoid disease		2.87% (2915)	3.11% (1781)	2.06% (718)	*
**Predictors**					
**Range**	**Measure**	Median [IQR]	Median [IQR]		
1–30 days	# of diagnoses	3 [0, 12]	3 [0, 13]	2 [0, 10]	*
1–30 days	# of lab results	0 [0, 46]	3 [0, 47]	0 [0, 43]	*
1–30 days	# of office visits	3 [1, 6]	3 [1, 6]	2 [1, 5]	*
1–30 days	# of emergency department visits	0 [0, 0]	0 [0, 0]	0 [0, 0]	*
1–30 days	# of hospitalizations	0 [0, 0]	0 [0, 0]	0 [0, 0]	*
1–365 days	# of diagnoses	15 [2, 51]	14 [2, 52]	11 [0, 36]	*
1–365 days	# of lab results	35 [0, 151]	34 [0, 142]	15 [0, 84]	*
1–365 days	# of office visits	11 [5, 25]	11 [5, 25]	9 [4, 20]	*
1–365 days	# of emergency department visits	0 [0, 1]	0 [0, 1]	0 [0, 1]	
1–365 days	# of hospitalizations	0 [0, 1]	0 [0, 1]	0 [0, 0]	*

*: Differences between training and testing sets are computed with: 1) χ2 tests for demographics; 2) proportion tests for individual comorbidities and mortality rates; and 3) Mann-Whitney tests for Charlson score and days from admission to death. In all cases, statistical significance is indicated (*) for adjusted *p* < 0.05 using a Bonferroni correction

^a^: Demographics coded within the EHR at the time of admission

^b^: Ethnicity contains many missing values which are omitted before computing the proportion and difference between groups

^c^: Including death and initiation of hospice care

^d^: Comorbidities are derived from ICD-10 diagnosis codes present in each patient’s year of history pre-admission using the diagnostic groups of the Charlson Comorbidity Index as implemented in the comorbidity R package [24]. Patients with no documented history are omitted from the denominator of each comorbidity

### Retrospective modeling

#### Performance within the training cohort

Sampling from the training set produced five comparable folds for cross-validation, each with a similar number of patients (14,230–14,617) and outcome rate (3.9–4.3%). AUROC and AUPRC within cross-validation from each model is reported in Table 2. The random forest classifier with 100 trees and a maximum depth of 1000 (eResults) outperformed the lasso regression model with marginal improvement over the XGBoost model and was selected as the final model (Table 2). The most influential predictors are aggregates that describe utilization (eTable 2).

**Table 2:** Model performance within cross-validation, applied to the testing set, and stratified by site

Model	Cohort	Measure	AUROC	AUPRC
Lasso regression	Training (Cross-validation)	Mean [min, max]	78.8 [78.0, 80.2]	21.0 [18.3, 22.0]
XGBoost	Training (Cross-validation)	Mean [min, max]	84.6 [83.8, 86.0]	25.7 [21.2, 27.4]
Random forest	Training (Cross-validation)	Mean [min, max]	86.9 [85.3, 87.7]	26.4 [20.1, 31.0]
	Testing (Bootstrapped)	Median [95% CI]	87.2 [86.1, 88.2]	28.0 [25.0, 31.0]
	Brooklyn	Median [95% CI]	83.8 [81.9, 85.6]	26.6 [22.5, 31.0]
	Non-Brooklyn	Median [95% CI]	**88.9** **[87.5, 90.2]**	**30.1** **[26.4, 33.7]**

#### Performance within the testing cohort

**Fig. 2:**
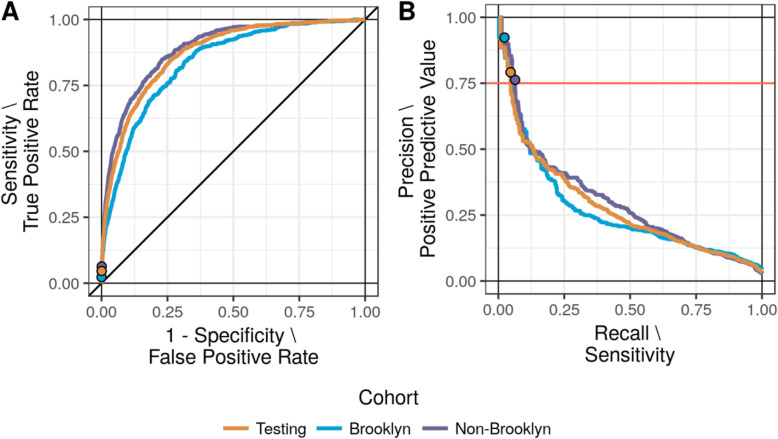
Performance curves from the complete testing cohort and further stratified by location. **a** receiver operating characteristic, and **b** precision-recall curves. The selected threshold is highlighted along with each corresponding point once stratified by location

When applied to the testing cohort, the random forest model performs similarly to cross-validation, with median and 95% confidence intervals (CIs) for AUROC and AUPRC reported in Table 2. The receiver operating characteristics and precision-recall curves of the entire testing set are described in Fig. 2 with the selected threshold highlighted. (Calibration is assessed in eResults and eFigure 3). Each category of data contributes to overall redundancy where removing any one has little to no effect on overall performance (eTable 3).

#### Performance across sites within the testing cohort

Table 2 also describes model performance within the Brooklyn sub-cohort, reporting a marginal decrease in AUROC, compared to the Non-Brooklyn group (combining Manhattan hospitals as both are faithfully represented). This divergence is visible within Fig. 2a. A similar pattern is observed in AUPRC, as described in Table 2 and Fig. 2b. Despite the visible region of decreased performance towards the bottom-left corner, the three curves of Fig. 2b overlap at PPV above 50% where our supportive care application is likely to operate. That is, the marginally worse global performance does not impact localized performance. (Calibration across locations is also assessed in eResults and eFigure 3).

#### Operating threshold within the testing cohort

A criterion for 75% PPV yields an operating threshold of 0.355 with 4.6% sensitivity/recall where patients exceeding this threshold are at very high risk of dying (eFigure 2B). Less than 5% of all deaths in the 60 days following admission can be predicted while maintaining this strict PPV constraint (no more than one false positive from four high-risk predictions). All-cause mortality includes entirely unpredictable deaths, increasing the denominator and shrinking sensitivity. This 4.6% group constitutes a real, potentially impactful group of patients at very high risk of dying.

Although the location-specific precision-recall curves of Fig. 2b overlap at 75% PPV, the corresponding thresholds are not guaranteed to be similar. Distributions of predicted probabilities across the three hospitals suggests high similarity between the two general hospitals (Brooklyn and Tisch) *except* in the very high-risk range (eFigure 4). When the operating threshold is applied to *only* Brooklyn patients, PPV shifts up to 92% as highlighted in Fig. 2b. The distributional differences between Brooklyn and non-Brooklyn populations combined with the imbalanced training proportions result in very precise, conservative application of the model to Brooklyn patients.

Application of one institution-wide threshold at Brooklyn poses little risk to patients but does perpetuate underrepresentation of Brooklyn patients. Several threshold-agnostic and threshold-specific measures of model performance are compared across locations and further across demographic sub-populations of sex, race, and ethnicity (eMethods, eResults, eFigures 5 and 6). Discrepancies in performance consistently under-represent Brooklyn patients at very high risk of dying but similar differences are observed across other demographics. Removal of race and ethnicity as features worsens these disparities. This bias further highlights the need for transparency and pragmatic solutions to reach equity.

### Prospective validation

Our model was silently tested for 12 weeks, beginning August 2018, by sending an once-a-day email to assess validity of patients above the operating threshold (and a sample below). The vast majority (78%; 74 of 95) of patients reviewed by a hospitalist were expected to benefit from supportive care. At the time of review, many of these patients were not, at least yet, receiving supportive care. Multiple comorbidities and complex disease were common, including patients who were not considered appropriate for intervention. During this period, a near real-time prediction system was developed to generate a prediction for each patient within minutes of admission.

After a successful *silent-live* period, the model was implemented *live* in October 2018. In the nine months through June 2019:

1. 49,785 inpatient admissions were detected for prediction,
2. 48,797 sets of data were collected from the database,
3. 41,728 predictions were made, where
4. 104 predictions exceeded the threshold.

Of the over forty thousand predictions, the median [IQR] time difference between admission and risk assessment was 1.3 [0.9, 32] minutes where 68% of predictions are made within five minutes of admission (database downtime causes delay where < 8% exceed six hours). Of 104 high-risk predictions, 27 were ill-timed (11 hospice admissions, 5 post-transplant admissions, and 11 encounters erroneously labeled ‘inpatient’). From the 77 well-timed predictions, 50 (65%) led to death or hospice within 60 days (median [IQR]: 25 [13, 61] days). Only 10 admissions (13%) have no known end-of-life outcome at last censor (median [IQR]: 250 [60, 292] days). Live application of our model has identified patients at very high risk of short-term death within minutes of admission.

## Discussion

### Prospective results and application feasibility

Prospective implementation of the final model produces a total of 41,728 predictions over nine months. The 75% PPV operating threshold identifies 104 admissions (0.25%) at very high risk. Although this proportion is small, it is consistent with other work and underscores how difficult it is to predict end-of-life with high confidence. The model is not perfect but neither is the standard of care. Many identified patients die within 60 days (65%) and may have benefited from earlier, more comprehensive discussions about their goals of care. Future work will assess the ability of physicians to recognize which identified patients will not die and the impact of predictions upon clinical intervention. The model and prediction system are working as designed and will be expanded into practice to recommend supportive care.

### Generalization to Brooklyn cohort

An evolving patient population is common in many applications and creates a practical challenge for prospective validation. In this case the mechanism of change is apparent: a new hospital was brought into the system that treats a new population with varying comorbidities and social determinants of health. Although this cause is obvious, the consequences are not. Along with an increase in proportion of patients observed at the Brooklyn hospital (38.0% in testing up from 9.5%; Table 1), there are corresponding structural differences in age, race, sex, outcome, and comorbidities between Manhattan and Brooklyn sites (eTable 1). These differences, indicative of a larger disparity between sites, further complicate generalization. Despite this, a model trained with only 10% of cases being from a new hospital can adapt to be performant and safely applied in a shifted patient mix.

The underrepresentation of Brooklyn patients during training does affect model performance at the Brooklyn hospital and their representation in the identified high-risk group (eFigures 5 and 6). A larger sample of Brooklyn patients for model training may improve the model’s ability to learn the new site and improve performance. Any potential risk to patient safety is mitigated as only patient-positive interventions [25] will be applied to identified patients with no change in care for unidentified patients. However, it is unfair to Brooklyn patients to concentrate the intervention and its benefits to Manhattan. One recent model fairness work [22] has suggested the use of multiple thresholds, one for each sensitive group. This concept resembles affirmative action, especially when intentionally used to help overcome a well-established social challenge discernible within data. A Brooklyn-specific threshold would ‘lower the bar’ for Brooklyn patients to the same predefined 75% PPV (or lower) in an attempt to encourage adoption and more widespread use of supportive care in that community. A 75% PPV threshold specific to Brooklyn was estimated at 0.295 which, when applied during the nine months of prospective validation, would have identified 450% more Brooklyn patients (55 vs. 10).

### Limitations

Not all aspects of generalizability or model fairness could be assessed in this work. Some aspects that need further assessment include: 1) data collection that may be different between locations, 2) generalization to similar patients in different geographic locations, 3) application of the model to sites that use different EHR technologies, 4) more formal statistical methods to model multiple sites.

## Conclusion

A machine learning model was developed and validated on retrospective patient data from three hospital sites. Assessment of model performance across sites and potentially sensitive demographics suggest varying degrees of unfairness as the one model and one operating threshold imperfectly learn differences in site, sex, race and ethnicity. Any degree of unfairness is ameliorated since the shift in performance at the underrepresented site raises the precision and sustains patient safety in the case of a patient-positive intervention. The model was implemented after initial testing reported the majority of cases were appropriate for intervention. When live, the model can deliver predictions within minutes of admission to prompt consideration by the care team and influence decision-making.

## Supplementary information

**Additional file 1.**

## Data Availability

The underlying protected health information is not available. High-level data, such as that described in the figures and tables herein, are available upon reasonable request only if sharing that data would not endanger anyone’s privacy.

## Abbreviations

AUPRC: Area under the precision-recall curve
AUROC: Area under the receiver operating characteristic
EHR: Electronic health record
IQR: Interquartile range
PPV: Positive predictive value
PRC: Precision-recall curve
ROC: Receiver operating characteristic
