# Supplementary material for: Development, implementation, and prospective validation of a model to predict 60-day end-of-life in hospitalized adults upon admission at three sites
Source: BMC Med Inform Decis Mak. 2020 Sep 7;20:214. doi: 10.1186/s12911-020-01235-6 (PMC7487547; doi:10.1186/s12911-020-01235-6)
Supplement: Supplementary file 1 — Additional file 1. [file 12911_2020_1235_MOESM1_ESM.pdf]

Supplementary Online Content

Development, Implementation, and Prospective Validation of a Model to Predict 60-day End-of-Life in Hospitalized Adults upon Admission at Three Sites

Vincent J Major, and Yindalon Aphinyanaphongs.

eMethods
1. Model Development
2. Operating Threshold
3. Evaluation in the Context of Potential Demographic Bias

eResults
1. Composite End-of-Life Outcome
2. Model Development
3. Evaluation in the Context of Potential Demographic Bias
    a. Testing Set Performance and Calibration
    b. Intersectional Subcohort Performance
    c. Explicit Removal of Demographics

eFigures
eFigure 1. Intersections between each of the three end-of-life outcome sources as an Upset chart.
eFigure 2. Survival curves stratified by development group, risk group and demographics.
eFigure 3. Calibration plots for the testing cohort and stratified by location.
eFigure 4. Distributions of uncalibrated predicted probabilities stratified by site.
eFigure 5. Global (threshold-agnostic) model performance within sensitive strata.
eFigure 6. Model errors within sensitive strata for the unmasked and masked models.
eFigure 7. Variable importance measured by selection frequency of each demographic feature in the final model.

eTables
eTable 1. Demographics, outcome, comorbidity, and model predictors of the testing set stratified by site.
eTable 2. Top 50 most selected model predictors.
eTable 3. Model performance after ablating one category of data.
eTable 4. Ethnicity and race masked model performance.
eTable 5. Demographics of identified patients by final and race and ethnicity masked models.

eReferences

This supplementary material has been provided by the authors to give readers additional information about their work.

eMethods

Model Development

Three alternative models were considered: 1. logistic regression with lasso regularization implemented with the *glmnet* package in R (1), 2. XGBoost with a logistic objective implemented with the *xgboost* package in R (2), and 3. random forest implemented in R using the *fest* program (3). Empirical testing of model parameters was conducted within 5-fold cross-validation within the training cohort where patients (4) are partitioned into five groups and five models are learnt, each leaving out a different fifth for validation. Different parameters are compared by computing the areas under the receiver operating characteristic (AUROC) and precision-recall curves (AUPRC) within each cross-validation fold and the mean AUROC and AUPRC across folds.

Operating Threshold

Given the predicted probabilities and known truths, a criterion is imposed to draw a single threshold that will separate predicted positives from predicted negatives. The metric and value used is application specific and depends on the 'cost' of both types of errors (5). Low cost interventions such as further diagnostic testing will greatly differ from a decision to perform costly treatment, for example. In this application, conservative identification of individuals at very high risk of near term death was the key objective as action will be taken only for those predicted at risk. Therefore, an operating criterion of 75% positive predictive value (PPV; otherwise known as precision) was selected—one false positive to three true positives.

To improve threshold robustness, 1000 bootstrap iterations are used to compute a median threshold. In each iteration, 80% of the test set is sampled (with replacement), a precision-recall curve created and a threshold selected at 75% PPV. The median threshold is then computed from the 1000 different values. This process adds robustness which is especially important at the very high PPV range as each false positive estimated at very high risk can greatly affect the path of the precision-recall curve at small cumulative samples.

Evaluation in the Context of Potential Demographic Bias

Given the demographic differences between development cohorts driven, in large, by structural differences across sites (observed in Table 1 and eTable 1), two experiments were conducted. First, as recommended by Mitchell et al. (6), an investigation of model performance in intersectional sub-cohorts of increasing complexity is conducted. AUROC and AUPRC are measures of global model performance and do not accurately describe performance at a particular threshold. To investigate differences in local performance across subpopulations, the procedure is repeated for false positive rate, false negative rate, false discovery rate and false omission rate.

Second, an otherwise identical model is developed while strictly removing 'sensitive' demographics of race and ethnicity and several likely proxies of religion and preferred language. Sex and age can also be considered sensitive demographics in applications outside healthcare (e.g. recidivism or lending). In this context of mortality risk it would be impractical to require equal, fair treatment across these groups and as such were not considered. To aid direct comparison, identical model parameters are used to replicate an identical training procedure.

These sensitive fields potentially help the classifier separate clusters of patients at different depths of the classifier's trees that subsequently improve learning. Accordingly, omission of these fields is expected to marginally reduce performance, at least in demographic sub-populations. To investigate any changes, an investigation of model performance in intersectional sub-cohorts is similarly performed for this 'masked' model. To investigate how the masked model compensates without proxies of race or ethnicity, feature importance of demographic predictors before and after masking is computed using selection frequency (7).

eResults

Composite End-of-Life Outcome

By combining the three available sources of patient outcomes (internal deaths, purchased deaths, and hospice discharges), 10,229 patient outcomes are discovered where 67% are affirmed by two or more sources (eFigure 1). The largest group of single-source outcomes is the hospice group where 45% of all patients discharged to hospice were subsequently lost to follow-up with no confirmed death or date of death (2,504 from 5,598). In the 3,094 admissions with both hospice and death outcomes, the median [IQR] time between discharge to hospice and death was 9 [4, 18] days. The addition of hospice adds some 'fuzziness' to the outcome but only for the 30% of end-of-life cases where the patient is discharged to hospice before death.

Model Development

Cross-validation across different parameter combinations found the random forest is relatively insensitive to parameters compared to the lasso regression and XGBoost alternatives. The random forest parameters with highest and most robust performance were 100 trees limited to a maximum depth of 1000. A final model was retrained on the entire training set with these parameters and applied to the temporally separated testing cohort. The most frequently selected predictors of the final model are reported in eTable 2.

Evaluation in the Context of Potential Demographic Bias

Testing Set Performance and Calibration

Within the entire testing set, the learned classifier has good performance (Table 2) and successfully separates patients by mortality risk (eFigure 2B) while being sufficiently well calibrated throughout the risk spectrum (eFigure 3A and B). The classifier also appears sufficiently well calibrated across locations (eFigure 3C and D), particularly Brooklyn and Tisch Hospitals, despite the demographic and outcome differences observed between sites (eTable 1). Of note, the classifier tends to underestimate mortality risk for patients within the top percentiles (observed mortality > estimated risk; intervals above the dashed diagonal line of eFigure 3) suggesting any selected threshold should conservatively maintain desired PPV.

Relatedly, the distributions of predicted probability within the testing set for the two general hospitals are remarkably similar only differing at the very high percentiles (eFigure 4; median [IQR] of Tisch vs Brooklyn 0.012 [0.0015, 0.044] vs 0.017 [0.0044, 0.046]). The Orthopedic hospital lags drastically with many fewer high risk patients (median [IQR], 0.00028 [0.00011, 0.0070]). These observations suggest a potential problem of infra-marginality that may challenge model fairness at any threshold (8).

Intersectional Subcohort Performance

To assess model fairness across sensitive demographics, global model performance (measured by AUROC and AUPRC) are compared across strata of sex, ethnicity and race as depicted as black intervals in eFigure 5A and B. Furthermore, each strata is further separated by location (Brooklyn or Non-Brooklyn, combining Tisch and Orthopedic hospital) to assess the divide caused by population differences and underrepresentation during training.

The reduced AUROC and AUPRC reported in Table 2 for the Brooklyn population are visible in almost all subpopulations of eFigure 5A and B with marginal exceptions of higher AUROC in Asian and Black patients and higher AUPRC in Black patients and men at Brooklyn. Of note, the Hispanic population at Brooklyn is likely under-labeled in the demographic data leading to smaller than expected sample sizes which lead to the wide confidence intervals observed.

A similar analysis performed at a specific threshold corresponding to 50% PPV (as sample size was too small for subpopulation analysis at our preferred 75% PPV), described similar patterns of performance differences in eFigure 6A–D. False positive and false discovery rates are lower for all Brooklyn patients and in subpopulations of men and White patients. False negative rate is higher for all Brooklyn patients and in subpopulations of women, White and Other Race patients. False omission rate is higher for all Brooklyn patients, for both men and women as well as

White patients. Unfortunately, the intersectional sample sizes limit more precise estimates especially for ethnicity and race subpopulations. Together these results suggest the site-specific differences and underrepresentation during training are causing the model to under-identify Brooklyn patients.

Explicit Removal of Demographics

The ‘masked’ model (trained on data with race, ethnicity and their proxies explicitly removed) results in model performance as described in eTable 4. Interestingly, the removal of race and ethnicity results in marginally improved testing set AUROC and AUPRC (comparing Table 2 and eTable 4) with little observable improvement across subpopulations (eFigure 5) with the exception of less variability in Hispanic patients at Brooklyn. When comparing error rates of the masked model to the unmasked model (eFigure 6), discrepancies between Brooklyn and Non-Brooklyn patients appear to be worsened across the board of false positive, false negative, and false omission rates. The explicit removal of sensitive predictors worsen the site-based disparity observed.

Exactly how the masking of race and ethnicity affects the classifier is difficult to determine. One may expect a shift in reliance from these predictors to other proxies of race or ethnicity. The selection frequency of demographic predictors used in the unmasked model (eFigure 7) describe the frequent use of each demographic including smoking status, sex, and age. When ethnicity, race, preferred language and religion are removed the selection frequency shifts randomly for the remaining demographics (‘X’ marks in eFigure 7) suggesting that none of these predictors are latched onto by the masked model. Comparing the top predictors of the unmasked and masked models in eTable 2 suggests little impact of masking race and ethnicity on these proxies of utilization where only 12 of 50 shift by more than ten places.

When similarly thresholded to a prespecified PPV of 75%, the two models identify a similar order of magnitude number of patients: 72 unmasked vs. 48 unmasked. However, there are only 31 patients in common. The ethnicity, race, sex, and location demographics of these patients are described in eTable 5. The masked model identifies fewer patients in total but the proportion of identified men and Asian patients increased (although the absolute number of men and Asian patients identified remains lower). Of note, the masked model does not improve the underrepresentation of Brooklyn patients.

eFigures

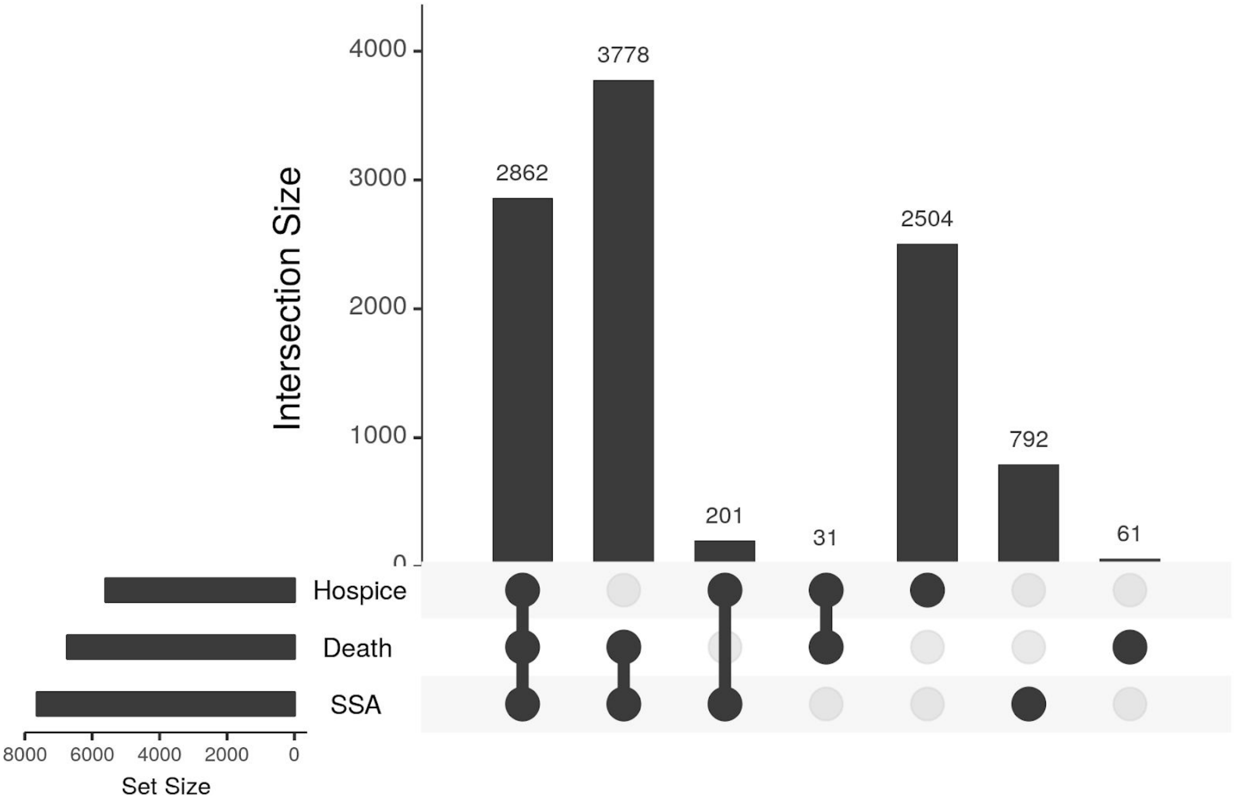

eFigure 1. Intersections between each of the three end-of-life outcome sources as an Upset chart.

Notes: Outcomes from all admissions within 2015–2017 are included here such that individual patients can be counted more than once. Hospice refers to discharge dispositions of either inpatient hospice or home hospice with the date of discharge used. Death refers to known dates of death internal to the EHR of varying upstream sources. SSA refers to the purchased data derived from the Social Security Administration's Master Death File.

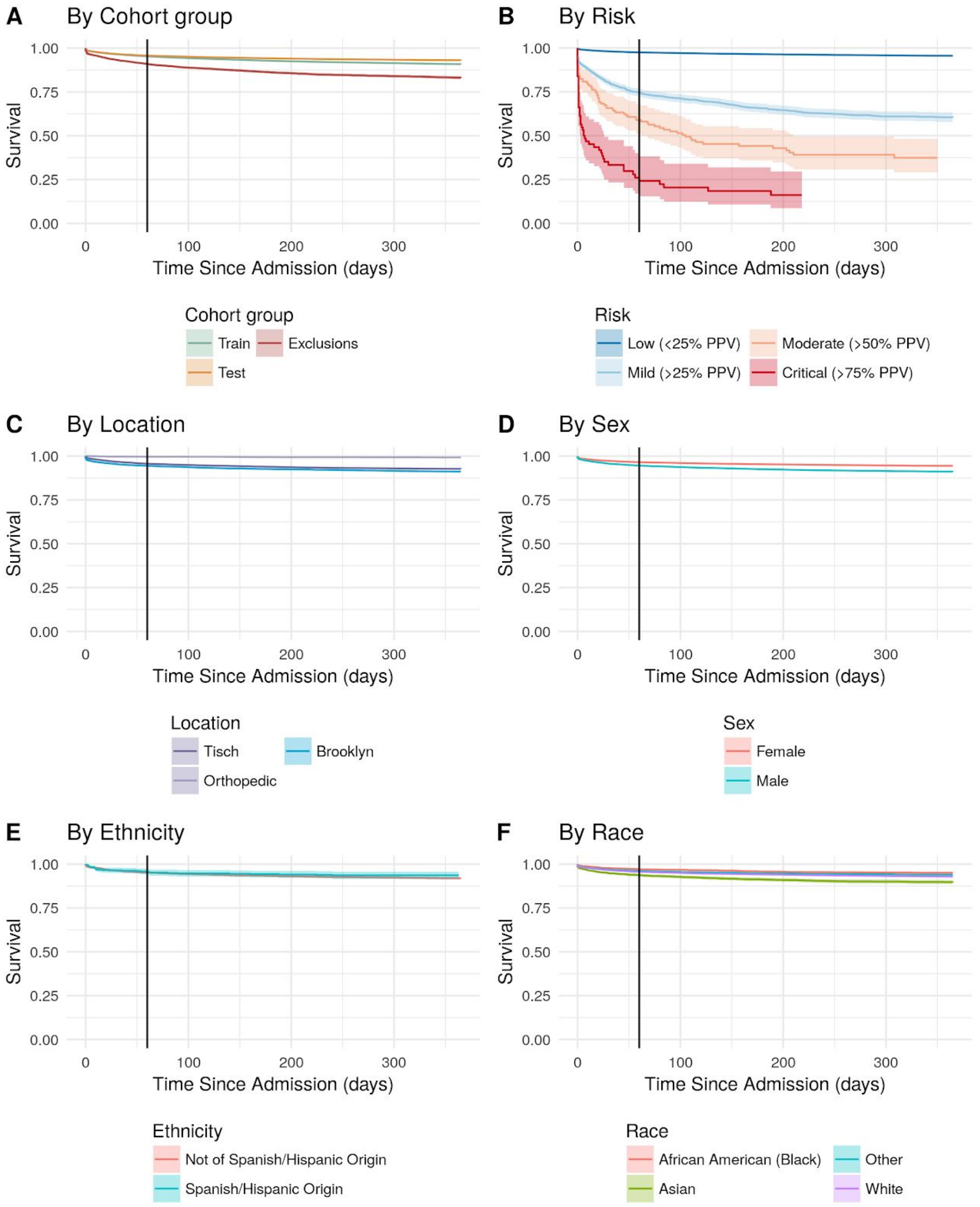

eFigure 2. Survival curves stratified by development group, risk group and demographics.
A) model development cohort as well subgroups of the testing cohort by: B) estimated risk group, C) location, D) sex, E) ethnicity and F) race.
Note: Risk groups are mutually exclusive such that the Moderate group consists of patients who did not exceed the threshold corresponding to 75% PPV but did exceed the one for 50% PPV. The unknown, other or patient refused options for sex, ethnicity and race were omitted for D) sex and E) ethnicity but collapsed into Other for F) race.

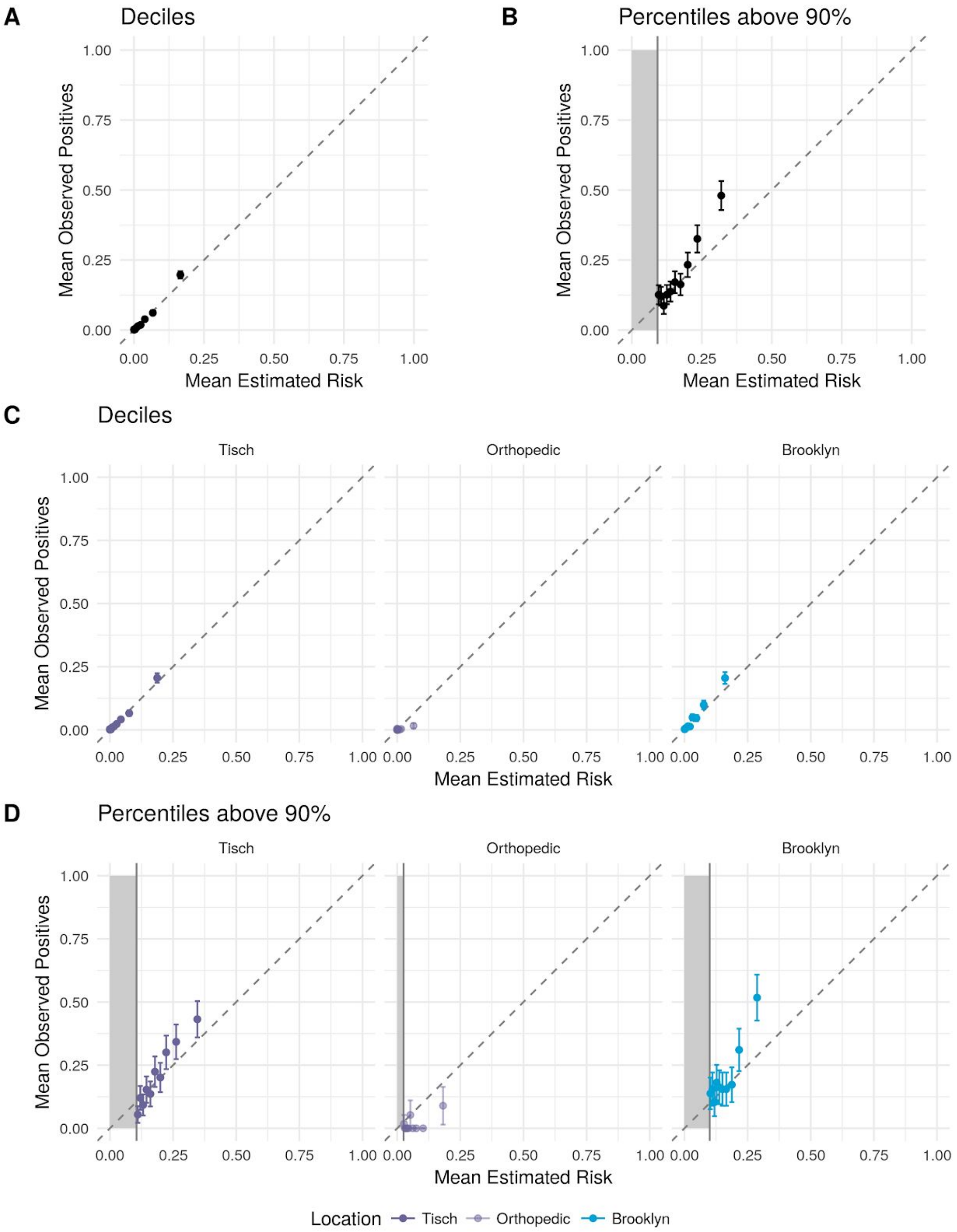

eFigure 3. Calibration plots for the testing cohort and stratified by location.

All testing patients A) by decile, and B) by percentile within the top decile of risk and C) by location and decile, and D) by location and percentile within the top decile.

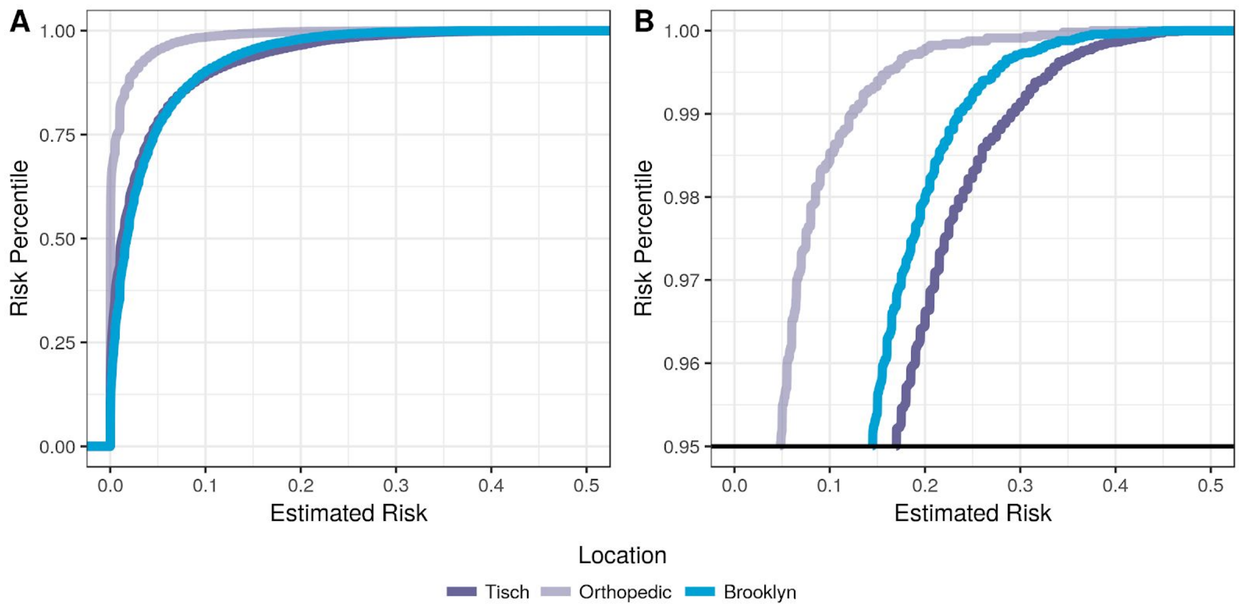

eFigure 4. Distributions of uncalibrated predicted probabilities stratified by site.
A) Global distributions and B) distributions within the top 5%.

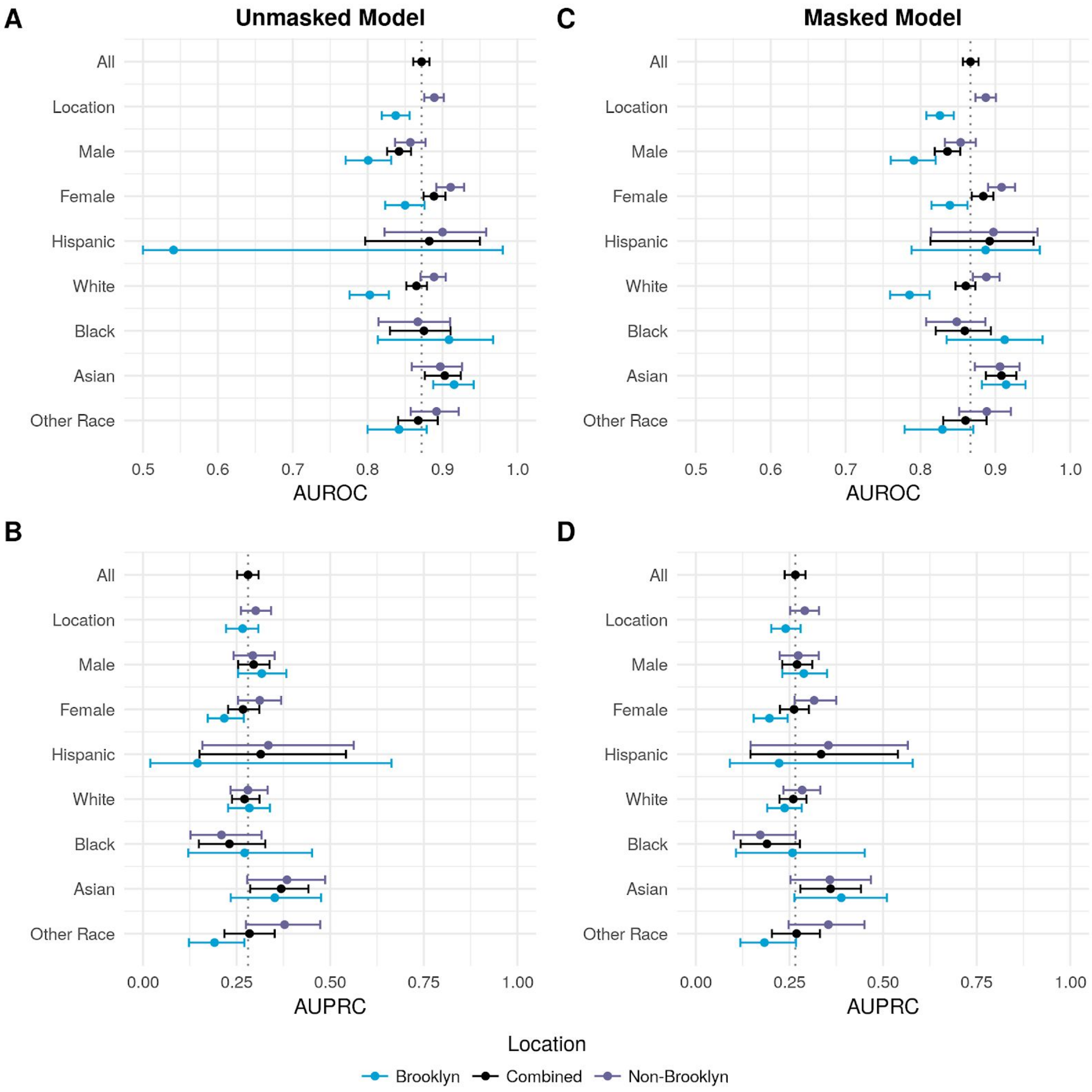

eFigure 5. Global (threshold-agnostic) model performance within sensitive strata for the unmasked and masked models.

Median [95% CI] AUROC and AUPRC within sub-cohorts of location, gender, ethnicity, and race.

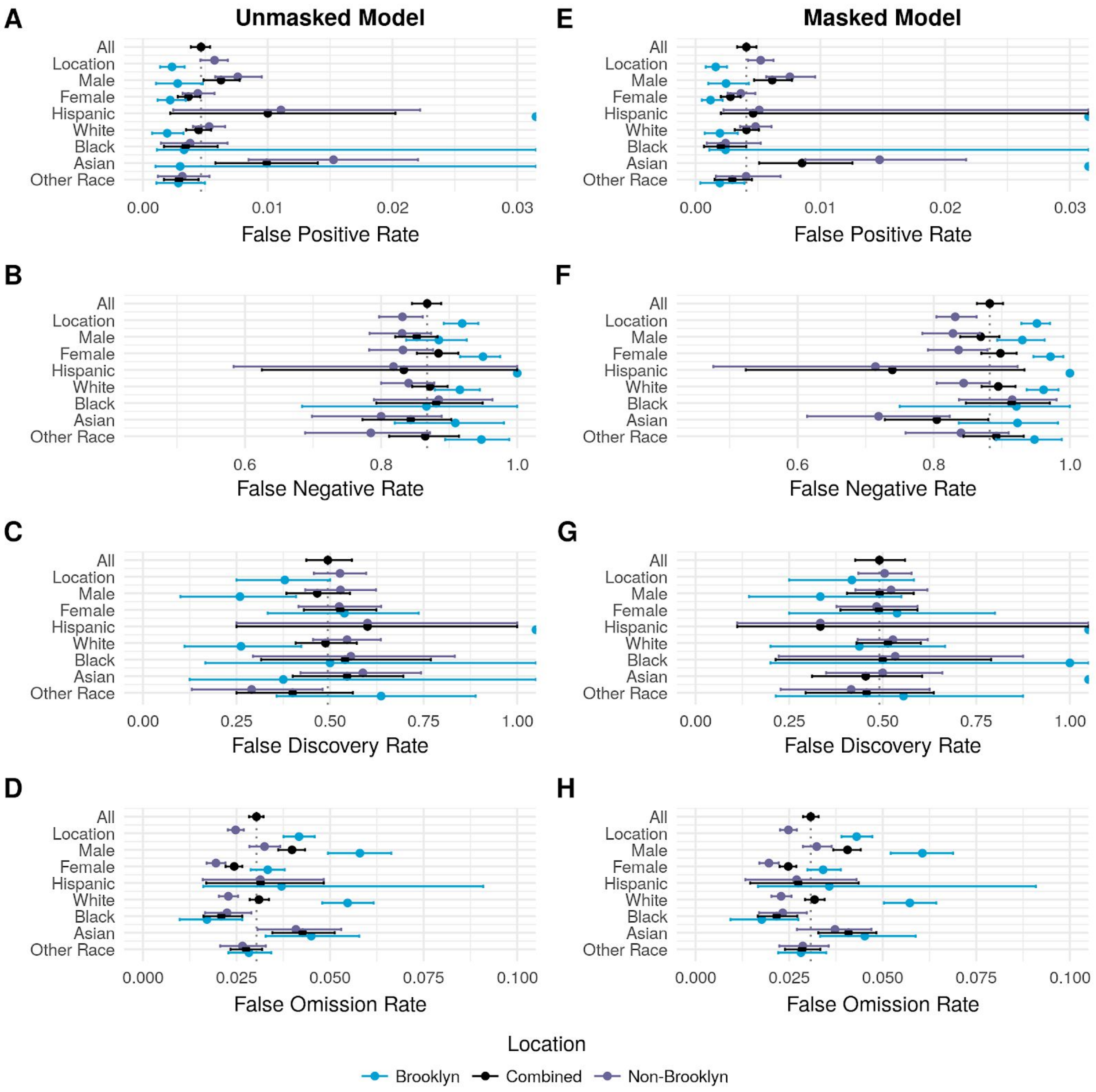

eFigure 6. Model errors within sensitive strata for the unmasked and masked models.
Median [95% CI] False Positive Rate, False Negative Rate, False Discovery Rate and False Omission Rate, each at a threshold corresponding to 50% PPV, within sub-cohorts of location, gender, ethnicity, and race.

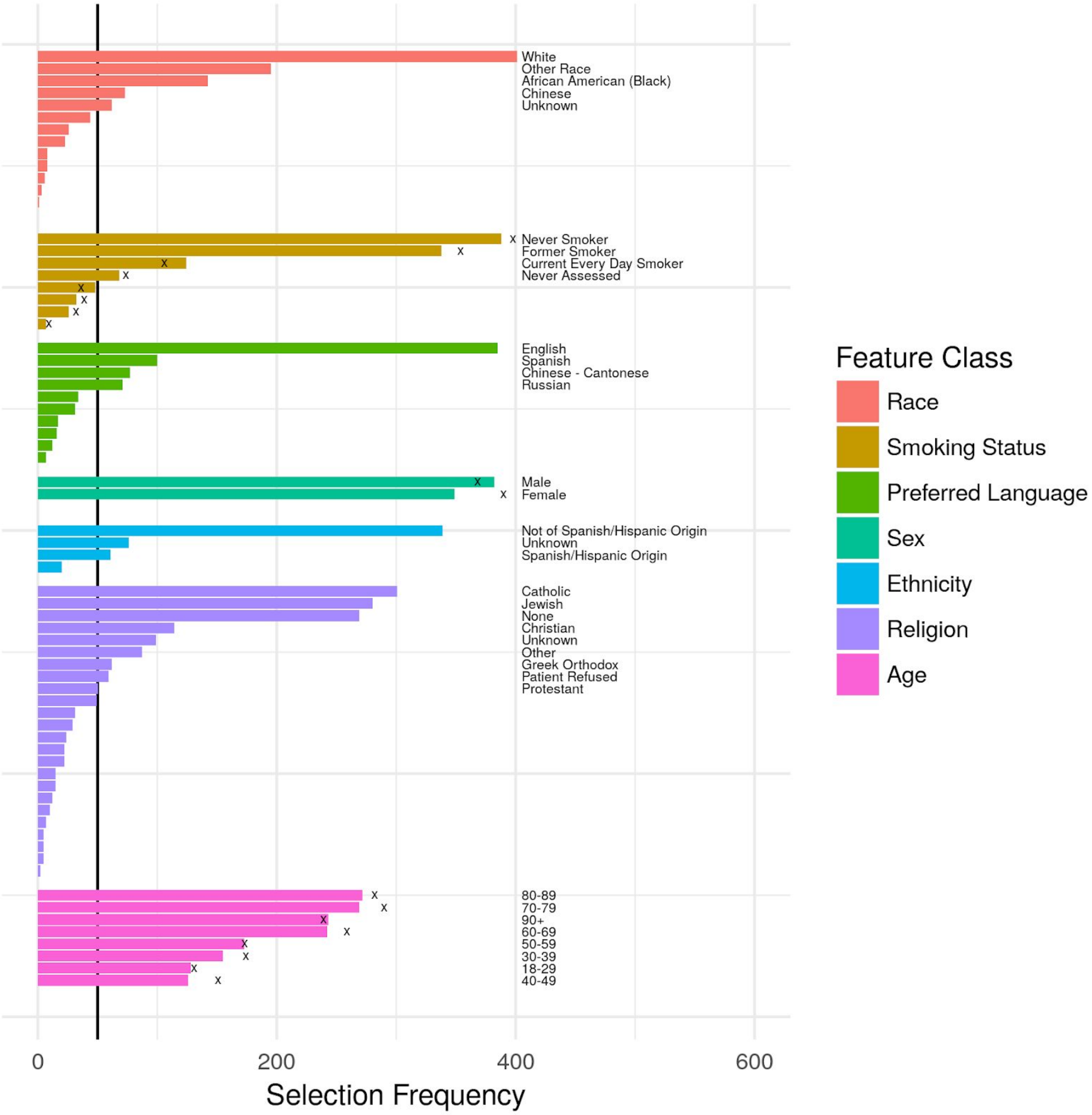

eFigure 7. Variable importance measured by selection frequency of each demographic feature in the final model. Points marked as 'X's indicate the shifted selection frequency after explicit masking of race, ethnicity, language and religion.

eTables

eTable 1. Demographics, outcome, comorbidity, and model predictors of the testing set stratified by site.
The Brooklyn hospital compared to the two non-Brooklyn locations combined.

			**All Testing Set Patients** n = 46,458	**Manhattan** n = 28,815	**Brooklyn** n = 17,643	
**Demographics** [a]						
	Measure	Value				
	Age		% (n)	% (n)	% (n)	*
		18-29	13.1% (6087)	10.2% (2949)	17.8% (3138)	
		30-39	18% (8361)	18.4% (5308)	17.3% (3053)	
		40-49	9.69% (4504)	9.67% (2785)	9.74% (1719)	
		50-59	13.4% (6206)	14.3% (4123)	11.8% (2083)	
		60-69	17.3% (8026)	19.4% (5583)	13.8% (2443)	
		70-79	15.1% (7008)	16.4% (4712)	13% (2296)	
		80-89	10.2% (4748)	9.21% (2655)	11.9% (2093)	
		90+	3.27% (1518)	2.43% (700)	4.64% (818)	
	Ethnicity [b]		% (n)	% (n)	% (n)	
		Hispanic	8.62% (666)	8.52% (589)	9.52% (77)	
		Not Hispanic	91.4% (7060)	91.5% (6328)	90.5% (732)	
		Unknown	-- (38732)	-- (21898)	-- (16834)	
	Race		% (n)	% (n)	% (n)	*
		Black	10.7% (4987)	11.3% (3248)	9.86% (1739)	
		East Asian	9.1% (4230)	6.07% (1750)	14.1% (2480)	
		West Asian	1.74% (807)	2.18% (627)	1.02% (180)	
		White	57.3% (26642)	64.8% (18682)	45.1% (7960)	
		Other	18.8% (8714)	13.2% (3814)	27.8% (4900)	
		Unknown	2.32% (1078)	2.41% (694)	2.18% (384)	
	Sex		% (n)	% (n)	% (n)	*
		Female	60.5% (28130)	59.2% (17063)	62.7% (11067)	
		Male	39.4% (18327)	40.8% (11751)	37.3% (6576)	
		Unknown	0% (1)	0% (1)	-	
	Site		% (n)	% (n)	% (n)	*
		Tisch	49.2% (22877)	79.4% (22877)	-	
		Orthopedic	12.8% (5938)	20.6% (5938)	-	
		Brooklyn	38.0% (17643)	-	100% (17643)	

			All Testing Set Patients n = 46,458	Manhattan n = 28,815	Brooklyn n = 17,643	
**Outcomes** [c]			% (n)	% (n)	% (n)	
	Any known death		3.57% (1657)	2.86% (824)	4.72% (833)	*
	60-day death		5.2% (2414)	4.34% (1252)	6.59% (1162)	*
			Median [IQR]	Median [IQR]	Median [IQR]	
	Days from admission to death		21 [1, 93]	27 [3, 104]	13 [1, 74]	*
**Comorbidities** [d]			Median [IQR]	Median [IQR]	Median [IQR]	
	Charlson Score		0 [0, 2]	0 [0, 2]	0 [0, 2]	*
			% (n)	% (n)	% (n)	
	AIDS/HIV		0.51% (176)	0.547% (129)	0.42% (47)	
	Cancer (any malignancy)		13.2% (4594)	15.3% (3609)	8.80% (985)	*
	Cerebrovascular disease		8.13% (2826)	7.60% (1792)	9.23% (1034)	*
	Chronic obstructive pulmonary disease		13.5% (4703)	12.1% (2858)	16.5% (1845)	*
	Congestive heart failure		8.56% (2978)	8.44% (1990)	8.82% (988)	
	Dementia		3.09% (1075)	1.96% (463)	5.46% (612)	*
	Diabetes with chronic complications		5.68% (1977)	4.35% (1025)	8.50% (952)	*
	Diabetes without chronic complications		14.4% (4995)	12.5% (2956)	18.2% (2039)	*
	Hemiplegia or paraplegia		2.35% (817)	2.08% (490)	2.92% (327)	*
	Metastatic solid tumour		4.55% (1584)	5.16% (1216)	3.29% (368)	*
	Mild liver disease		5.14% (1787)	5.11% (1205)	5.20% (582)	
	Moderate or severe liver disease		1.11% (385)	1.31% (310)	0.67% (75)	*
	Myocardial infarction		6.90% (2400)	6.21% (1465)	8.35% (935)	*
	Peptic ulcer disease		1.27% (443)	1.17% (275)	1.50% (168)	
	Peripheral vascular disease		9.97% (3469)	10.6% (2510)	8.56% (959)	*
	Renal disease		7.93% (2759)	7.24% (1708)	9.38% (1051)	*
	Rheumatoid disease		2.06% (718)	2.31% (545)	1.54% (173)	*
**Predicted Risk**			% (n)	% (n)	% (n)	
	Any risk		76.7% (35620)	83.3% (24003)	65.8% (11617)	*
	High-risk	75% Positive Predictive Value	0.20% (72)	0.25% (59)	0.11% (13)	
	High-risk	50% Positive Predictive Value	0.91% (323)	1.06% (254)	0.59% (69)	*

Notes:
*: Differences between Manhattan and Brooklyn patients within the testing set are computed with: 1) $\chi 2$ tests for demographics; 2) proportion tests for individual comorbidities, mortality rates and predicted risk groups; and 3) Mann-Whitney tests for Charlson score and days from admission to death. In all cases, statistical significance is indicated (*) for adjusted $p < 0.05$ using a Bonferroni correction.
a: Demographics coded within the EHR at the time of admission.
b: Ethnicity contains many missing values which are omitted before computing the proportion and comparing between groups.
c: Including death and initiation of hospice care.
d: Comorbidities are derived from ICD-10 diagnosis codes present in each patient's year of history pre-admission using the

diagnostic groups of the Charlson Comorbidity Index as implemented in the comorbidity R package (9). Patients with no documented history are omitted from the denominator of each comorbidity.

eTable 2. Top 50 most selected model predictors.

Comparing the final model and the experimental 'masked' model with race and ethnicity omitted. Highlights describe absolute rank differences greater than 10.

Final model			Masked model	
**Rank**	**Selection Frequency**	**Predictor**	**Rank**	**Selection Frequency**
1	656	Maximum # of diagnosis codes per day	2	620
2	641	Unique # of diagnosis codes	1	669
3	625	Mean # of diagnosis codes per day	4	606
4	619	Mean # of office visits per day	8	587
5	600	Total # of office visits	7	596
6	596	Unique # of offices visited	5	599
7	587	Mean # of diagnosis codes per day	10	566
8	561	Total # of diagnosis codes	3	617
9	559	Office visits at 'NYU LANGONE HEALTH'	6	597
10	554	Total # of diagnosis codes	**31**	492
11	549	Maximum # of diagnosis codes per day	15	539
12	543	Unique # of offices visited	11	562
13	541	Maximum # of office visits per day	13	557
14	539	Total # of office visits	**27**	497
15	537	Mean # of office visits per day	12	559
16	537	Office visits at 'NYU LANGONE HEALTH'	19	522
17	530	Total # of office visits	9	571
18	530	Office visits at 'NYU LANGONE HEALTH'	14	554
19	525	Mean # of lab results per day	34	485
20	523	Unique # of diagnosis codes	16	537
21	520	Total # of diagnosis codes	26	503
22	512	Mean # of diagnosis codes per day	**36**	482
23	499	Maximum # of diagnosis codes per day	21	512
24	493	Office visit of type 'Appointment'	25	504
25	492	Mean # of diagnosis codes per day	33	485
26	490	Unique # of offices visited	18	524
27	489	Maximum # of administered medications per day	43	466
28	489	Unique # of nonsurgical procedures	41	468
29	488	Unique # of diagnosis codes	38	480

30	483	Mean # of office visits per day	**42**	467
Final model			Masked model	
**Rank**	**Selection Frequency**	**Predictor**	**Rank**	**Selection Frequency**
31	480	Total # of diagnosis codes	**44**	466
32	479	Minimum # of lab results per day	40	475
33	477	Total # of lab results	23	507
34	476	Range over # diagnosis codes each day	**56**	424
35	476	Total # of nonsurgical procedures	29	493
36	474	Mean # of nonsurgical procedures per day	**47**	461
37	469	Minimum # of diagnosis codes per day	**17**	525
38	467	Maximum # of office visits per day	48	460
39	467	Office visits at 'NYU LANGONE HEALTH'	45	465
40	465	Maximum # of diagnosis codes per day	20	512
41	464	Office visits with appointment length '15'	37	481
42	461	Unique # of diagnosis codes	32	490
43	459	Maximum # of office visits per day	51	432
44	459	Unique # of offices visited	**30**	493
45	452	Maximum # of lab results per day	**24**	506
46	452	Total # of office visits	**35**	484
47	451	Unique # of lab results	**22**	510
48	445	Mean # of office visits per day	46	463
49	438	Office visit of type 'Appointment'	49	460
50	435	Variance of # of office visits per day	50	440

eTable 3. Model performance after ablating one category of data.
Performance of the final model when applied to five testing sets each with a different category of data removed.

Cohort	Ablation	Measure	AUROC	AUPRC
Testing	None (Table 2)	Median [95% CI]	87.2 [86.1, 88.2]	28.0 [25.0, 31.0]
	Encounters	Median [95% CI]	86.0 [85.6, 86.5]	23.3 [22.1, 24.4]
	Diagnoses	Median [95% CI]	85.8 [85.3, 86.3]	24.0 [22.9, 25.3]
	Procedures	Median [95% CI]	87.1 [86.6, 87.6]	27.2 [25.9, 28.5]
	Medications	Median [95% CI]	87.2 [86.7, 87.7]	27.8 [26.6, 29.2]
	Lab Results	Median [95% CI]	86.4 [85.9, 86.8]	24.5 [23.2, 25.7]

eTable 4. Ethnicity and race masked model performance.
Performance within cross-validation, when applied to the test set and stratified by Brooklyn or not from the test set.

Cohort		Measure	AUROC	AUPRC
Cross-validation		Mean [min, max]	88.0 [87.2, 88.7]	27.7 [25.8, 31.0]
Testing		Median [95% CI]	88.2 [86.7, 89.8]	29.5 [24.4, 34.1]
	Brooklyn	Median [95% CI]	84.6 [82.3, 87.2]	27.4 [21.3, 34.2]
	Non-Brooklyn	Median [95% CI]	**90.0 [88.1, 92.0]**	**32.3 [25.2, 38.1]**

eTable 5. Demographics of identified patients by final and race and ethnicity masked models.

		**Final model** (n=72)	**Masked model** (n=48)
Ethnicity	Hispanic	2.78% (2)	0% (0)
Race	Asian	20.8% (15)	29.2% (14)
	Black	6.94% (5)	6.25% (3)
	White	55.6% (40)	47.9% (23)
	Other Race	15.3% (11)	16.7% (8)
Sex	Female	50% (36)	39.6% (19)
	Male	50% (36)	60.4% (29)
Location	Brooklyn	18.1% (13)	18.8% (9)
	Non-Brooklyn	81.9% (59)	81.2% (39)

eReferences

1. Friedman J, Hastie T, Tibshirani R. Regularization paths for generalized linear models via coordinate descent. J Stat Softw [Internet]. 2010;33(1):1. Available from: https://www.ncbi.nlm.nih.gov/pmc/articles/pmc2929880/

2. Chen T, Guestrin C. XGBoost: A Scalable Tree Boosting System. In: Proceedings of the 22nd ACM SIGKDD International Conference on Knowledge Discovery and Data Mining [Internet]. New York, NY, USA: Association for Computing Machinery; 2016 [cited 2020 Aug 13]. p. 785–94. (KDD '16). Available from: https://doi.org/10.1145/2939672.2939785

3. Karampatziakis N. FEST: fast ensembles of sparse trees [Internet]. 2009. Available from: lowrank.net/nikos/fest/

4. Neto EC, Pratap A, Perumal TM, Tummalacherla M, Snyder P, Bot BM, et al. Detecting the impact of subject characteristics on machine learning-based diagnostic applications. npj Digital Medicine [Internet]. 2019 Oct 11 [cited 2020 Jan 15];2(1):1–6. Available from: https://www.nature.com/articles/s41746-019-0178-x

5. Turney PD. Cost-sensitive classification: Empirical evaluation of a hybrid genetic decision tree induction algorithm. J Artif Intell Res [Internet]. 1994;2:369–409. Available from: http://www.jair.org/papers/paper120.html

6. Mitchell M, Wu S, Zaldivar A, Barnes P, Vasserman L, Hutchinson B, et al. Model cards for model reporting. In: Proceedings of the conference on fairness, accountability, and transparency [Internet]. 2019. p. 220–9. Available from: https://dl.acm.org/doi/abs/10.1145/3287560.3287596?casa_token=3PBkTTlsmtcAAAAA:nNzj1Iarxtqlz88MX6jtKk2c5Vv9qHXSo10OXt77M_DfO1ydx1zAkH_AKMt-mEQmeoQRjWyWxJCAeA

7. Weng SF, Reps J, Kai J, Garibaldi JM, Qureshi N. Can machine-learning improve cardiovascular risk prediction using routine clinical data? PLoS One [Internet]. 2017 Apr 4;12(4):e0174944.

Available from: http://dx.doi.org/10.1371/journal.pone.0174944

8. Corbett-Davies S, Goel S. The Measure and Mismeasure of Fairness: A Critical Review of Fair Machine Learning [Internet]. arXiv [cs.CY]. 2018. Available from: http://arxiv.org/abs/1808.00023

9. Gasparini A. comorbidity: An R package for computing comorbidity scores. Journal of Open Source Software [Internet]. 2018;3(23):648. Available from: https://joss.theoj.org/papers/10.21105/joss.00648.pdf
